# Comparative Efficacy and Safety of Antifungal Agents in the Prophylaxis of Oropharyngeal Candidiasis among HIV-Infected Adults: A Systematic Review and Network Meta-Analysis

**DOI:** 10.3390/life12040515

**Published:** 2022-03-31

**Authors:** Shamala Gopal Rajadurai, Mari Kannan Maharajan, Sajesh K. Veettil, Divya Gopinath

**Affiliations:** 1 School of Postgraduate Studies, International Medical University, Kuala Lumpur 57000, Malaysia; shamala.g@moh.gov.my; 2 Department of Pharmacy Practice, School of Pharmacy, International Medical University, Kuala Lumpur 57000, Malaysia; marikannan@imu.edu.my; 3 Department of Pharmacotherapy, College of Pharmacy, University of Utah, Salt Lake City, UT 84112, USA; sajesh.veettil@pharm.utah.edu; 4 Clinical Oral Health Sciences, International Medical University, Kuala Lumpur 57000, Malaysia

**Keywords:** oropharyngeal candidiasis, oral candidiasis, HIV, antifungal agents, prevention, prophylaxis, systematic review, network meta-analysis

## Abstract

The objective of the study was to compare the efficacy and safety of antifungal agents used in the prevention of oropharyngeal candidiasis among HIV-infected adults. A systematic search was conducted in four databases (MEDLINE, Scopus, CENTRAL, and Embase) for eligible randomized control trials (RCTs). The network meta-analyses (NMA) were performed using a random-effects model. Interventions were ranked based on the efficacy and safety using the surface under the cumulative ranking curve (SUCRA). The quality of evidence was assessed using the GRADE approach. From a total of 1574 studies screened, 7 RCTs comprising 959 participants were included in NMA. The use of fluconazole as a prophylactic agent was associated with a significant reduction in incidence of OPC compared to placebo (RR, 0.45 (95% CI: 0.27–0.77)) in HIV-infected adults. The overall quality of evidence was graded as moderate. Fluconazole was ranked the best antifungal for efficacy (SUCRA—95.6%) as well as safety (SUCRA—39.3%) in HIV-infected adults. Overall, the quality of evidence was graded as moderate. Fluconazole can be considered as an effective agent with a better safety profile for the prophylaxis of OPC in HIV-infected adults. However, similar to any other antimicrobial agent, the risk of possibility of resistance must be weighed against the benefits.

## 1. Introduction

Oropharyngeal candidiasis (OPC) is attributed to the overgrowth of the commensal fungi, *Candida* spp., in the mouth and throat. *Candida* spp. is commonly found in the gastrointestinal tract of humans [1]. OPC is commonly seen in individuals with compromised immune systems, including those with diabetes, HIV infection, or those receiving chemotherapy [2,3,4,5,6].

Among HIV-infected adults, there is an increased risk of developing OPC due to the loss of cell-mediated immunity. Furthermore, HIV-infected patients are also affected by hyposalivation, reduced salivary anticandidal proteins, and other physiological changes that may alter the oral microbiota and increase the risk of OPC [7]. Moreover, multiple deficiencies in the host defense mechanism might also play a major role. The detailed pathogenesis underlying the predisposition of HIV-infected patients for OPC is unclear. The most common forms of OPC in the HIV-infected population include pseudomembranous, erythematous, and angular cheilitis, and it may develop at any point of the disease timeline [6,7,8].

OPC is projected to affect at least one-third of the HIV-infected adults [7,8,9], and 90% of HIV patients will likely experience this opportunistic infection during their period of the illness. An increase in the prevalence of OPC among HIV patients is linked to lower levels of CD4+ cells. However, in the early stages of HV infection, oral *Candida* spp. carriage is greatly enhanced, regardless of the patient’s CD4+ cell count [6,7,8]. However, as the condition persists, the frequency of esophageal and pseudomembranous candidiasis has been shown to be increasingly common. It remains the most frequently encountered oral infection in HIV patients [10,11] and negatively impacts the quality of life (QoL) of this population [12]. Prophylaxis for the prevention of opportunistic infections has been suggested as an integral element for the management of HIV-infected adults and has been shown to reduce HIV-associated mortality among patients with low CD4+ counts (<50 cell/cm^3^) [13]. Nevertheless, few other investigators have suggested that routine use of secondary prophylaxis for OC is only advised when patients suffer from severe or frequent recurrent infections [14,15,16]. The role of antifungal prophylaxis among immunocompromised patients has been highlighted by Meunier et al. [14]. Few studies have reported the clinical benefits of antifungals in preventing the occurrence of OPC infections among HIV patients [1,3,14,15,16]. Both itraconazole and fluconazole have been found to be effective in preventing the recurrence of OC [14,16]. However, there are currently no available direct comparisons in terms of efficacy between the two drugs. When compared to fluconazole, both nystatin and clotrimazole were found be less effective in preventing OC [14,15,16]. The use of antifungal prophylaxis has been suggested to be considered in the event where the patient suffers from severe and recurrent OPC [17]. The previous published systematic review on this topic was conducted in 2001 by Patton et al. [18]. There are many available interventions for the prevention of OPC, and choosing a drug is a great challenge in clinical practice. 

Uncertainty remains in the comparative effectiveness and safety of available antifungal agents for the prevention of OPC in HIV-infected patients. There has not been any direct comparison of the efficacy and safety of different antifungal agents used in the prevention of OPC in HIV-infected adults, and hence, the evidence is still inconclusive. Conducting a network meta-analysis would allow for both direct and indirect comparisons of the different antifungal agents used in the prevention of OPC, and the network estimates derived can assist the health care providers and policy makers in decision making [19]. Hence, the objective of our paper is to review the published studies systematically and to perform a network meta-analysis to compare the efficacy and the safety profile of the different antifungal agents used prophylactically in the prevention of OPC in HIV-infected patients.

## 2. Materials and Methods

This study was registered on PROSPERO with the registration number CRD42020202356. The systematic review and network meta-analysis were carried out in accordance with the Cochrane Handbook for Systematic Reviews of Interventions [20], and the results are reported following the Preferred Reporting Items for Systematic Reviews and Meta-Analyses (PRISMA) extension statement for NMA [21]. The review addresses the following research questions regarding the prevention of OPC in HIV-infected adults:

1. Which antifungal agent is more effective in the prevention of OPC in HIV-infected adults according to the evidence from randomized controlled trials (RCTs)?
2. Which antifungal agent used for the prevention of OPC among HIV-infected adults has a better safety profile according to the evidence from RCTs?

### 2.1. Search Strategy and Study Selection

Four databases (MEDLINE, CENTRAL, Scopus, Embase) were thoroughly searched for randomized controlled trials involving antifungal agents used for the prevention of OPC among HIV-infected adults. The search included studies that were published up to 15 October 2020. We also conducted a manual search of published systematic reviews to identify more related studies. Search was confined to the English language.

The detailed search strategy is provided in the supplementary material (Supplementary Tables S1 and S2).

Studies included were RCTs that met the following inclusion criteria:

(i) HIV-infected patients as subjects,
(ii) intervention including any class of antifungal agent used to prevent OPC,
(iii) comparison against placebo, any other antifungal agent, or no treatment,
(iv) primary outcome as the incidence of OPC and the development of adverse effects as the secondary outcome.

Studies that did not report the outcomes, conference abstracts, non-RCT observational studies, and retrospective studies were excluded.

### 2.2. Data Extraction and Quality Assessment

Data extraction was done independently by two reviewers (S.G.R., D.G). Any disagreements among reviewers were sorted out with a third party. Published findings from each of the studies identified were extracted and further classified into the following sections: study characteristics, population characteristics, intervention characteristics, and outcomes. Information regarding the adverse effects of each drug and comparator was extracted from each study and categorized. The Cochrane risk-of-bias tool for randomized trials (RoB 2) [22] was utilized to assess the risk of bias among the studies that were included in this review. Each investigator performed the analysis separately, and conflicts were discussed and decided via a third party. The Grading of Recommendations, Assessment, Development, and Evaluation (GRADE) approach was also used to rate the quality of evidence (high, moderate, low, and very low) of estimates derived from NMA [23].

### 2.3. Data Synthesis and Statistical Analysis

The initial number of participants randomly assigned to each trial arm was used to perform the analysis for all outcomes (intention-to-treat principle) [21]. Hence, study participants who were lost to follow-up were considered to have not developed OPC. The outcomes were expressed as the risk ratio with a 95% CI. For direct comparison, a standard, pairwise meta-analysis was conducted with a random-effects (DerSimonian and Laird) model [21,24]. If a direct comparison was based on two or more studies, heterogeneity between trials was assessed by considering the I^2^ statistics; an I^2^ estimate of ≥50% was interpreted as evidence of substantial levels of heterogeneity [24]. The random-effects network meta-analysis (NMA) was performed with a consistency model, and the network inconsistency was assessed using the design-by-treatment inconsistency model [25]. The effectiveness of each antifungal agent was ranked based on the lowest rate of OPC relapse, and its safety was based on the lowest rate of total adverse effects reported according to their surface under the cumulative ranking (SUCRA) score [26]. Publication bias was assessed using a comparison-adjusted funnel plot, and all statistical analyses were conducted with STATA version 15.0 (StataCorp, College Station, TX, USA). An additional sensitivity analysis was performed using low-risk bias trials to assess the robustness of the primary outcome.

## 3. Results

### 3.1. Study Selection

**Figure 1:**
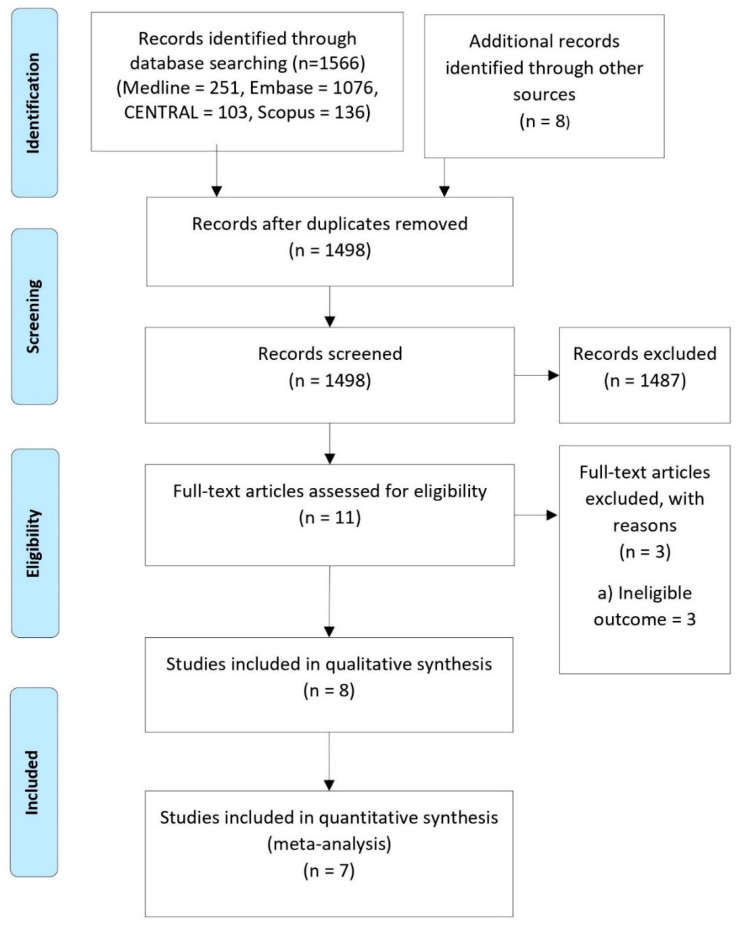
PRISMA flow diagram for selection of studies.

Four databases were searched for relevant randomized controlled trials (MEDLINE = 251, Embase = 1076, CENTRAL = 103, Scopus = 136). Manual searching was done on published reviews to identify any studies that were potentially missed out. From a total of 1574 studies, 1563 were excluded after duplicate removal and title and abstract screening. The remaining 11 studies were subjected to full-text screening, and an additional 3 studies [15,27,28] were excluded as they reported ineligible outcomes (Table S3). Eight studies were included for qualitative analysis and seven for quantitative analysis. The PRISMA flow diagram is as shown in Figure 1. 

### 3.2. Study Characteristics

The quantitative analysis of the efficacy and safety of the different antifungal agents used as prophylaxis of OPC among HIV-infected adults included seven [16,29,30,31,32,33,34,35] out of the eight identified studies. The 7 trials included 959 participants, from which 6 trials compared fluconazole to placebo [29,30,31,32,33,34,35] and 1 trial compared itraconazole to placebo [16]. The study characteristics of these studies are shown in Table 1.

### 3.3. Risk of Bias

**Figure 2:**
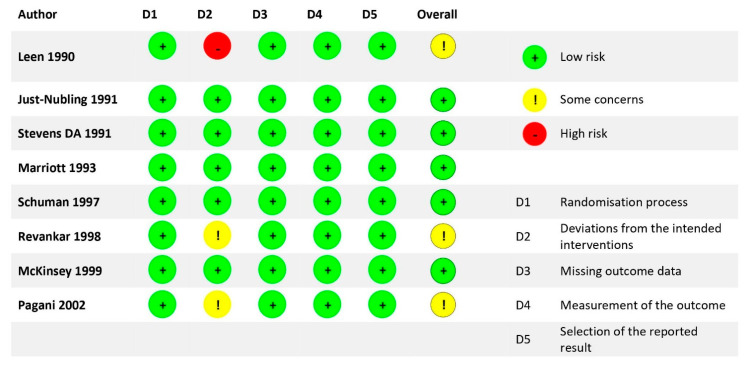
Risk of bias for selected studies.

The risk of bias is presented in Figure 2. Three [29,34,35] out of the eight studies had some concerns of risk of bias, while the remaining five studies had a low risk of bias. The concerns of risk of bias were mainly related to deviations from intended intervention as the data.

### 3.4. Efficacy of Antifungal Agents Used in the Prevention of OPC

**Figure 3:**
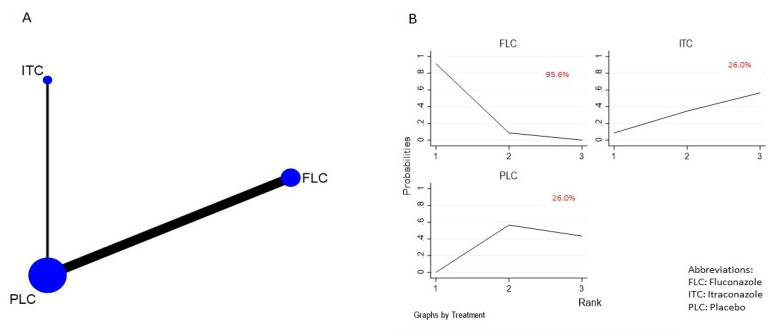
(**A**) Network plot comparing the efficacy of antifungal agents used for the prophylaxis of OPC among HIV-infected adults. (**B**) SUCRA ranking curve of antifungal agents used for the prophylaxis of OPC among HIV-infected adults (efficacy).

**Figure 4:**
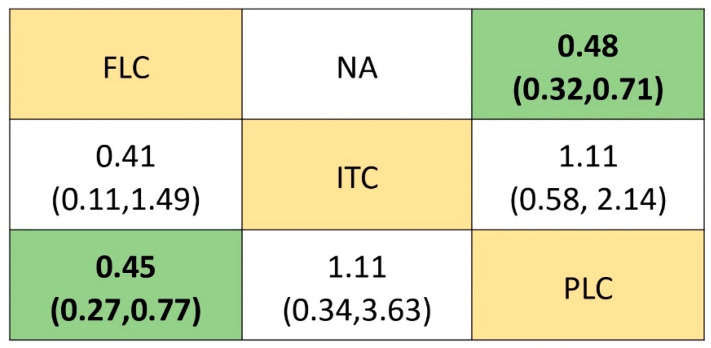
Comparative efficacy of antifungal agents used for the prophylaxis of OPC among HIV-infected adults. Abbreviations: FLC: fluconazole; ITC: itraconazole, PLC: placebo. Note: Pairwise (upper right portion) and network (lower left portion) meta-analytic results. Outcomes are expressed as risk ratio (95% confidence intervals). For the pairwise meta-analyses, a relative risk of less than 1 indicates that the treatment specified in the row is more efficient. For the network meta-analysis, a relative risk of less than 1 shows that the treatment specified in the column is more efficient. Bold and shaded results indicate statistical significance. NA indicates that there is no direct comparison to show the effect size.

The NMA of the efficacy of the different antifungal agents used as prophylaxis of OPC among HIV-infected adults included seven [16,29,30,31,32,33,34,35] out of the eight identified studies. The 7 trials included 959 participants, from which 6 trials compared fluconazole to placebo and 1 trial compared itraconazole to placebo. The network plot representing this relationship is shown in Figure 3A. In the network plot, each node represents a treatment arm, the size of the node indicates the total number of participants in that intervention, and the thickness of the connecting lines corresponds to the number of trials included. The ranking of the treatment options was analyzed via the surface under the cumulative ranking curve (SUCRA). The higher-ranking antifungal agent would be the one with the higher SUCRA score. Based on the analysis, fluconazole was ranked the highest (SUCRA score = 0.956) (Figure 3B). The NMA suggested that fluconazole is effective in preventing the occurrence of OPC among HIV-infected adults when compared to (RR, 0.45 (95% CI: 0.27–0.77)) placebo. However, no statistically significant difference was observed when compared to itraconazole (RR, 0.41 (95% CI: 0.11–1.49)). Itraconazole was not effective when compared to placebo (RR, 1.11 (95% CI: 0.34–3.63)). These results are summarized in the league table (Figure 4) and Supplementary Table S4. Fluconazole remained significant and ranked first even after a sensitivity analysis performed on trials with a low ROB (Supplementary Table S5). 

**Figure 5:**
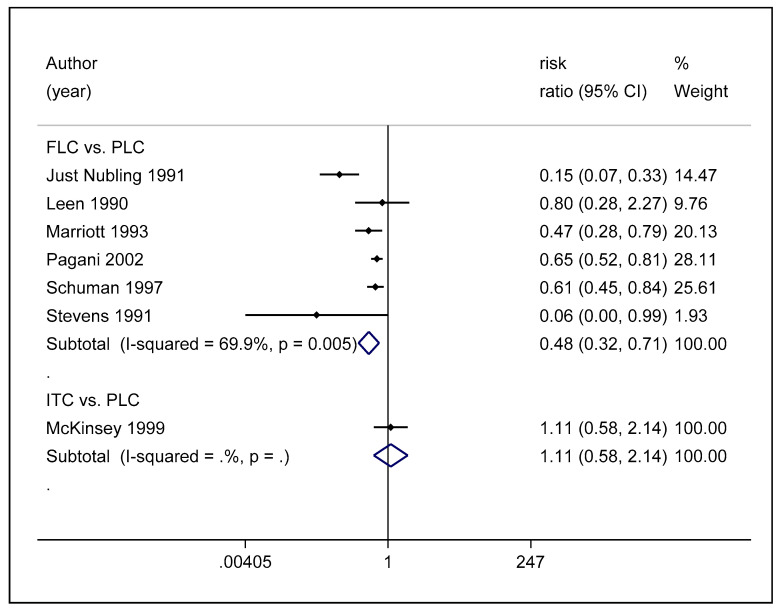
Forest plot of pairwise meta-analysis of antifungal agents used for the prevention of OPC among HIV-infected adults.

A pairwise meta-analysis was conducted on six studies that compared the efficacy of fluconazole to placebo in preventing the occurrence of OPC among HIV-infected adults. This analysis reports that fluconazole is capable of reducing the occurrence of OPC among HIV-infected adults compared to placebo, and its effect is statistically significant (RR, 0.48 (95% CI: 0.32–0.71)) (Figure 5). However, the heterogenicity was high between the studies (*p* = 0.005, I^2^ = 69.9%). There was only one study that compared the efficacy of itraconazole to that of placebo, and there were no significant differences between them in preventing the occurrence of OPC among HIV adults. Figure 5 depicts the forest plot of the pairwise meta-analysis of antifungal agents used to among HIV-infected adults for the prevention of OPC. 

### 3.5. The Safety Profile of Antifungal Agents Used in the Prevention of OPC among HIV-Infected Adults

From of the 8 articles that were reviewed for the prevention of OPC, 10 adverse effects were identified. These included general adverse effects such as an increase in liver enzymes, fever, night sweats, joint pain, neck swelling, adenopathy, and headache. Other reported effects were classified into gastrointestinal adverse effects, respiratory adverse effects, and dermatological adverse effects. The detailed prevalence of these has been tabulated and presented in Supplementary Tables S6–S10.

**Figure 6:**
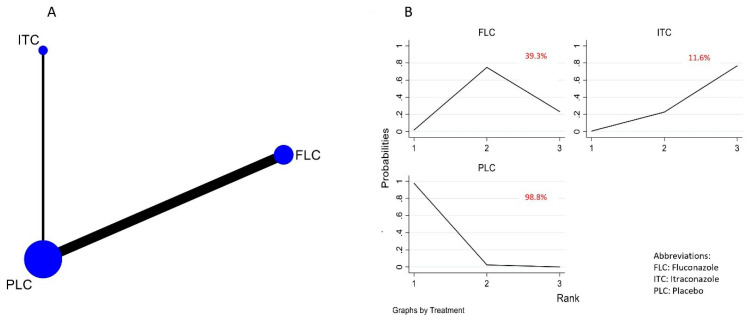
(**A**) Network plot comparing the safety of antifungal agents used for the prophylaxis of OPC among HIV-infected adults. (**B**) SUCRA ranking curve of antifungal agents used for the prophylaxis of OPC among HIV-infected adults (safety).

**Figure 7:**
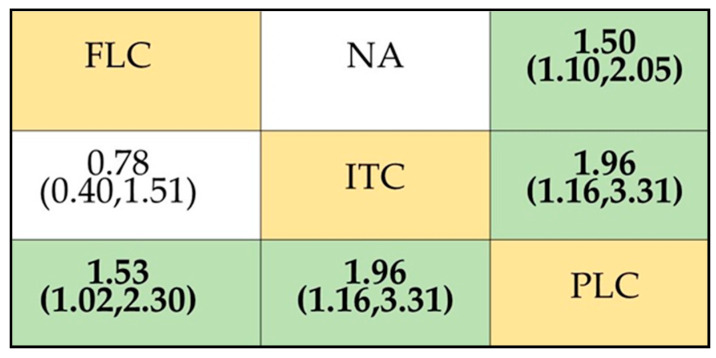
Comparative safety of fluconazole and itraconazole in preventing OPC. Abbreviations: FLC: fluconazole; ITC: itraconazole, PLC: placebo. Note: Pairwise (upper right portion) and network (lower left portion) meta-analytic results. Outcomes are expressed as risk ratio (95% confidence intervals). For the pairwise meta-analyses, a relative risk of less than 1 indicates that the treatment specified in the row is safer. For the network meta-analysis, a relative risk of less than 1 shows that the treatment specified in the column is safer. Bold and shaded results indicate statistical significance. NA indicates that there is no direct comparison to show the effect size.

**Figure 8:**
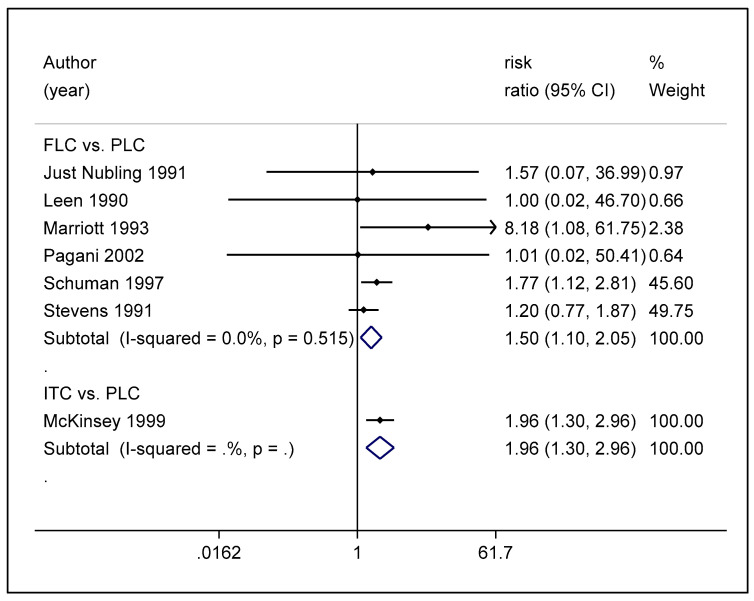
Forest plot of pairwise meta-analysis of the safety of antifungal agents used for the prevention of OPC among HIV-infected adults. Abbreviations: PLC—placebo, FLC—fluconazole, ITC—itraconazole.

The quantitative analysis of the safety of antifungals used in preventing OPC among HIV-infected adults was defined by the total number of adverse events reported for each treatment arm in the seven studies that were subjected to network meta-analysis. The network meta-analysis (Figure 6A) illustrated that fluconazole is associated with a 53% greater risk of the development of adverse effects compared to placebo (RR, 1.53 (95% CI: 1.02–2.29) (Figure 7). However, though safer than itraconazole (RR 0.78 (95% CI: 0.40–1.51)), the difference observed was not statistically significant (Figure 7). Itraconazole was associated with a 96% greater risk of the development of adverse effects compared to placebo (RR 1.96 (95% CI: 0.40–1.51)), and hence, when the ranking in terms of the safety of antifungal agents according to SUCRA, fluconazole was ranked higher for safety (Figure 6B). These results are presented in Figure 6 and Supplementary Table S11. The pairwise meta-analysis also illustrated similar findings (Figure 8).

### 3.6. Network Consistency and Small Study Effects

The assessment of the overall inconsistency for the solutions in the primary network analysis and sensitivity analysis is shown in Supplementary Table S12. A global inconsistency test by fitting design-by-treatment in the inconsistency model showed no inconstancy for the outcome in both primary and sensitivity analyses. The comparison-adjusted funnel plots for efficacy and safety are provided in Supplementary Figures S1 and S2 and indicate publication bias.

### 3.7. Grade Quality Assessment

Overall, the quality of evidence for fluconazole to prevent OCP in comparison to placebo was graded as moderate. There were three comparisons, with two direct and one indirect comparison. The quality of the network estimate for fluconazole vs. placebo was graded as high quality (RR, 0.45 (95% CI = 0.27–0.77)), while for itraconazole vs. placebo it was RR, 1.11 (95% CI = 0.34–3.63) and for fluconazole vs. itraconazole it was RR, 0.41 (95% CI = 0.11–1.49). Grading of these comparisons is provided in table Supplementary Table S13. The indirect estimates for fluconazole vs. placebo and itraconazole vs. placebo could not be generated using the node-splitting technique, as there were no triangular or quadratic loops present in the network.

## 4. Discussion

Due to the different levels of immunodeficiency encountered by HIV patients in their course of disease, determining optimal therapeutic options for secondary infections is difficult. One of the most significant tactics for achieving the aforementioned goal has always been prevention of secondary infections, which can deteriorate the quality of life and occasionally lead to life-threatening situations.

Even though the prevalence of OPC has dropped off after the introduction of highly active antiretroviral therapy (HAART), it remains a dilemma for clinicians and oncologists. OPC has been suggested as a valuable biomarker for HIV disease progression owing to antiretroviral therapy (HAART) failure as the prevalence of OPC directly correlates with the HIV viral load [36,37]. Within the HIV population, the use of antifungal interventions often helps in reducing clinical symptoms, thereby delivering a transient clinical response by reducing the number of fungi in the affected area [18,38]. However, complete eradication of the *Candida* spp. can be challenging, and as the HIV infection proceeds, the patients tend to experience more relapses and shorter disease-free intervals. Therefore, for HIV patients with frequent occurrences of OPC, secondary prophylaxis may be beneficial; however, there is concern regarding the issue of azole resistance secondary to long-term exposure to fluconazole. 

In this systematic review and network meta-analysis, we combined direct and indirect evidence to compare antifungal prophylaxis options for OPC in HIV-infected adults. To the best of our knowledge, this is the first network meta-analysis to report the pooled comparative efficacy of available medical interventions on the prevention of OPC episodes in the HIV-infected population. We present the pooled data of 959 patients incorporating trials with fluconazole and itraconazole compared to placebo. On analysis, itraconazole was found to be not effective in preventing the occurrence of OPC, while fluconazole, on the other hand, was able to achieve a 55% relative risk reduction in OPC episodes when compared to placebo. The overall quality of evidence according to GRADE for this comparison was found to be moderate. The findings of our study can be considered as an update for the evidence for the Cochrane review published in 2010 by Pienaar et al. [39], which however had utilized combined data for esophageal and oropharyngeal candidiasis; our focus was exclusively on oropharyngeal candidiasis.

The published RCTs are heterogenous in terms of drug dosage regimens used for the prophylaxis. Just-Nübling et al. compared two different fluconazole doses (50 and 100 mg) with a control group who did not receive any secondary prophylaxis. The cases included were HIV-infected patients with a CD4 lymphocyte cell count less than 100 cells/mm^2^ [30]. The patients who received fluconazole prophylaxis had significantly had fewer relapses of OPC compared to those who did not receive it (*p* < 0.01), and there was no difference in terms of the number of relapses between the two doses of fluconazole [30]. Thus, the study suggested that low-dose fluconazole, 50 mg once a day, could prevent OPC recurrence and be beneficial to HIV-infected patients who are in the advance stages. Another randomized, double-blind placebo study was conducted on 25 patients who were diagnosed with HIV by Stevens et al. to assess the benefit of 100 mg daily of fluconazole in preventing OPC recurrence [31]. Stevens et al. reported no cases of OPC relapse in the arm that received fluconazole as compared to 8 out of 13 patients in the placebo arm for the study duration of 12 weeks [31]. This supported the findings that were published by Just-Nübling et al., although the sample size was small (*n* = 25). 

Weekly fluconazole dosing regimens have been studied to see whether they may be beneficial as secondary prophylaxis for OPC. Leen et al. studied the benefits of fluconazole at a dose of 150 mg weekly compared to placebo, and they reported that the majority of those in the weekly fluconazole arm (5 out of 9) were free of OPC relapse for the study duration of 24 weeks, while all 5 of the patients in the placebo group had relapsed [33]. Similar studies were conducted by Marriot et al. [32] and Pagani et al. [34]. These studies compared the effectiveness of a 150 mg weekly dose of fluconazole to that of placebo with a larger sample size than the previous study. Marriot et al. conducted a randomized double-blind study on 84 HIV-infected patients and found that the risk of relapsing was reduced by 56% when compared to the placebo group, and this reduced risk of OPC relapse was significant (95% CI, 0.29–0.66) [32]. Marriot et al. also reported that there was a significantly lower rate of mycological relapse among those who received fluconazole prophylaxis compared to those in the placebo group (*p* = 0.004) [32]. Pagani et al. reported that relapse of OPC was experienced in 61% of those who received fluconazole compared to 90% of those in the placebo group (*p* < 0.001) [34]. In this trial, it was also reported that the time to the primary end-point, defined as the third OPC relapse, was significantly longer among patients who received weekly fluconazole prophylaxis compared to that in those who received placebo (*p* = <0.0001) [34]. 

Guidelines have not recommended any primary and secondary prophylaxis for mucosal candidiasis; however, studies have suggested that appropriate antiretroviral therapy can prevent the occurrence of OPC [17,40]. Another reason behind the concern of whether to prescribe antifungals for prophylaxis is the emergence of azole resistance. Pagani et al. analyzed the development of resistance to fluconazole among the study participants and reported that weekly doses of fluconazole as secondary prophylaxis did not have any significant impact [34].A similar study conducted by Revankar et al. also assessed the risk of fluconazole resistance among those who received daily doses of fluconazole along with rate of OPC relapse among HIV-infected patients [35]. They reported that 25% of patients who received fluconazole daily experienced events of OPC relapse as compared to 82% of those who did not; added to that, there was no significant difference in the resistance rates between the two groups, suggesting that daily use of fluconazole as secondary prophylaxis does not increase the risk of developing resistant strains [35]. 

Fluconazole, which belong to the family of azoles, mainly acts by inhibiting the cytochrome P450 enzyme lanosterol demethylase (14α-demethylase), encoded by *ERG11*, in the ergosterol biosynthesis pathway [40,41]. Ergosterol is an indispensable component of fungal membranes, and cell growth is arrested because of this inhibition of the synthesis. Several resistance mechanisms have been noted in different candidal species, mainly mutations involving *EGR11* and others, loss of heterozygosity, and changes in ploidy. Loss-of-function mutations in *ERG3* in *C. albicans* and *C. dubliniensis* have been associated with drug resistance to azoles [41]. Rosana et al. reported the combined overexpression of *CDR2* and *ERG11* as well as the presence of a mutation in the *ERG11* gene to be associated with fluconazole resistance in *C. albicans* from HIV patients [42]. Evidence with regards to azole resistance in the literature is conflicting as two studies linked the presence of azole-resistant strains of *C. albicans* among HIV patients with low CD4 cell count with prolonged prior exposure to fluconazole [43,44], while other RCTs have shown no significant differences in terms of the emergence of azole resistance when fluconazole was given continuously as prophylaxis as compared to intermittent dosing [34,35].

The analysis for the safety profile of antifungal agents used in preventing OPC in this study ranked fluconazole higher than itraconazole. Fluconazole has been known to be less likely to cause hepatotoxicity and to have better tolerability when compared to itraconazole [45]. Another important aspect to be taken into consideration would be the drug interactions, which are unavoidable in HIV patients owing to the coexistence of epidemics of HIV, tuberculosis, and other opportunistic infections [46]. Most tuberculosis drugs, including rifampicin, are potent inducers of cytochrome P450 enzymes. It has been reported that the concomitant administration of rifampicin with fluconazole modifies the pharmacokinetic factors of fluconazole, including a significant increase in its elimination rate constant resulting in shorter elimination half-life [47]. Moreover, the concomitant use of rifampicin with ketoconazole and itraconazole reduced the serum concentrations of these drugs [48]. Hence, careful consideration is required in such exceptional situations. Moreover, it is important to evaluate and report the prevalence of OPCs in HIV patients regularly, especially in middle- and low-income countries, which will make the decision to use prophylaxis straightforward. A strong and compelling benefit is essential when prophylaxis is weighed against the unavoidable complications with regards to drug interactions and adverse effects [48].

An important limitation of our meta-analysis is the lack of studies comparing the prophylactic effectiveness of other newer antifungal agents available in the market. No new studies are available in the literature with newer drugs such as posaconazole and echinocandins, which are reported to have a better safety profile than the older antifungal agents [49]. The studies available in the literature have been published in early 2000 or before. This may be partly due to the fact OPC is no longer a significant problem in developed countries, where major RCTs has been conducted until now. However, it is crucial to recognize that OPC still remains a substantial challenge for HIV patients in the developing world, which reiterates the persistent inequity in health outcomes around the world [50]. Evident gaps persist in the prevalence of different oral manifestations in HIV, including OPC, in some countries in Asia Africa and South America [50,51]. This might be due to the fact that the accessibility to HAART continues to be considerably less in the developing countries when compared to developed countries [50,52]. The recent workshop in Bali, Indonesia, in September 2019 also advocated that OPC continues to be a challenging oral manifestation of HIV, highlighting the importance of continuing the research in the field [50,51]. Furthermore, though many studies suggest ‘HAART’ to be effective in reducing the prevalence of opportunistic infections, several other reports have shown that patients with poor compliance to these medications have thrice the risk of developing any opportunistic infection in comparison to those with good compliance [53,54,55], and hence, patient compliance to HAART can be considered as the chief determining factor regarding opportunistic infections.

Secondly, the comparison-adjusted funnel plot indicates that there is a presence of publication bias. Moreover, there was a lack of RCTs examining OPC prevention in children, and hence, our review and meta-analyses were confined to HIV-infected adults. Among the RCTs we reviewed, the number of participants in the studies ranged from 24 to 323, with 5 studies reporting less than 100 participants. The secondary outcome measures investigated varied among the trials making it challenging to pool the results. Further trials adhering to the recommend CONSORT guidelines will improve research, reporting, and hence clinical practice. If predictive tools for the clinical progression monitoring of HIV patients who would benefit from prophylaxis can be developed, it can help in the selection of the subset of high-risk patients for the same.

## 5. Conclusions

The findings from this NMA show that fluconazole is beneficial in the prevention of OPC in HIV-infected adults. However, the use of fluconazole as secondary prophylaxis should be weighed against the cost, possible drug–drug interactions, and drug resistance, which may arise from the routine use of fluconazole as secondary prophylaxis. Further studies should be conducted to identify the optimal parameters for the use of antifungals for the prevention of OPC. High-quality trials are needed to compare fluconazole with relevant new comparators’ prevention as well as other outcomes, including adverse effects and quality of life. Future work should also focus on the cost-effectiveness of use of antifungals for the prevention of OPC.

## Figures and Tables

**Table 1:** Characteristics of the selected studies in details.

Author, Year	Country	No. of Participants	Study Design	Study Comparison	Outcome (Incidence of OPC)
Leen et al., 1990 [29]	UK	24	RCT	Weekly Fluconazole vs. Placebo	FLC: 4/12 PLC: 5/12
Just- Nübling et al., 1991 [30]	Germany	65	RCT	Daily Fluconazole vs. Untreated	FLC: 6/43 UT: 20/22
Stevens et al., 1991 [31]	USA	25	RCT	Daily Fluconazole vs. Placebo	FLC: 0/12 PLC: 8/13
Marriott et al., 1993 [32]	NM	84	RCT	Weekly Fluconazole vs. Placebo	FLC: 13/44 PLC: 25/40
Schuman et al., 1997 [33]	NM	323	RCT	Weekly Fluconazole vs. Placebo	FLC: 42/162 PLC: 68/161
Revankar et al., 1998 [35]	USA	62	RCT	Daily Fluconazole vs. Intermittent Fluconazole	Daily FLC: 4/20 Intermittent FLC: 23/42
McKinsey et al., 1999 [16]	USA	295	RCT	Itraconazole vs. Placebo	ITC: 17/149 PLC: 15/146
Pagani et al., 2002 [34]	Switzerland	143	RCT	Weekly Fluconazole vs. Placebo	FLC: 41/71 PLC: 64/72

Abbreviations: NM, not mentioned; RCT, randomized control trial; FLC, fluconazole; PLC, placebo; UT, untreated; ITC, itraconazole.

## Data Availability

Not applicable.

## Supplementary Materials

The following supporting information can be downloaded at https://www.mdpi.com/article/10.3390/life12040515/s1: Table S1: Search strategy Table S2: Search algorithm Table S3: Studies excluded from quantitative analysis Table S4: SUCRA rank of antifungal agents in the prevention of OPC among HIV-infected adults. Table S5: SUCRA rank of antifungal agents in the prevention of OPC among HIV-infected adults (sensitivity analysis). Table S6: General adverse effects reported (prevention of OPC) Table S7: Gastrointestinal adverse effects reported (prevention of OPC) Table S8: Respiratory adverse effects reported (prevention of OPC) Table S9: Dermatological adverse effects reported (prevention of OPC) Table S10: Incidence of elevated liver enzymes reported (prevention of OPC) Table S11: SUCRA rank of the safety of antifungal agents used for the prevention of OPC among HIV-infected adults. Table S12: Inconsistency in network meta-analysis.Table S13: Grade quality assessment. Figure S1: Comparison-adjusted funnel plot of antifungal agents used for the prevention of OPC among HIV-infected adults (efficacy), Figure S2: Comparison-adjusted funnel plot of interventions used for the prevention of OPC among HIV-infected adults (safety profile).
