# Supplementary material for: Comparative Efficacy and Safety of Antifungal Agents in the Prophylaxis of Oropharyngeal Candidiasis among HIV-Infected Adults: A Systematic Review and Network Meta-Analysis
Source: Life (Basel). 2022 Mar 31;12(4):515. doi: 10.3390/life12040515 (PMC9025400; doi:10.3390/life12040515)
Supplement: Supplementary file 1 [file life-12-00515-s001.zip › life-1618692-supplementary.pdf]

life

MDPI

Systematic Review

Comparative Efficacy and Safety of Antifungal Agents in the Prophylaxis of Oropharyngeal Candidiasis among HIV-Infected Adults: A Systematic Review and Network Meta-Analysis

Shamala Gopal Rajadurai [1], Mari Kannan Maharajan [2], Sajesh K. Veettil [3] and Divya Gopinath [4,*]

[1] School of Postgraduate Studies, International Medical University, Kuala Lumpur 57000, Malaysia; shamala.g@moh.gov.my
[2] Department of Pharmacy Practice, School of Pharmacy, International Medical University, Kuala Lumpur 57000, Malaysia; marikannan@imu.edu.my
[3] Department of Pharmacotherapy, College of Pharmacy, University of Utah, Salt Lake City, UT 84112, USA; sajesh.veettil@pharm.utah.edu
[4] Clinical Oral Health Sciences, International Medical University, Kuala Lumpur 57000, Malaysia
* Correspondence: divyagopinath@imu.edu.my

Supplementary Materials:

Table S1. Search strategy.

**Review question**	**In HIV-Infected Adults, Which Antifungal Treatment is More Effective and Safer to Prevent OPC?**
**Population**	HIV-infected adults receiving prophylaxis for OPC
**Sub-group**	If heterogenicity is present:<br>1. Drug doses<br>2. Dosing frequency
**Intervention**	Any intervention which is meant to prevent OPC, including systemic and topical antifungal agents, traditional medication, and other interventions.
**Comparison**	Placebo, no treatment, or any intervention against those interventions mentioned above (including different doses of the same intervention).
**Outcomes**	***Primary outcome:*** incidence of relapse of OPC<br>***Secondary outcome:*** adverse events ('probably due to drug') if mentioned by the study.
**Study design**	RCTs or Systematic reviews
**Databases**	Medline, Scopus, Embase, CENTRAL

Table S2. Search algorithm for Medline, Embase, CENTRAL and Scopus.

**Database**		**Query**	**Items Found**
Medline on Ovid	1. HIV	exp HIV/ or HIV.mp. OR HIV Infections.mp. or exp HIV Infections/ OR hiv-1.mp. or exp HIV-1/ OR hiv-2.mp. or exp HIV-2/ OR human immunodeficiency virus.mp. or exp HIV/ OR human immune-deficient virus.mp. OR (human immun*.mp. AND deficiency virus.mp.) OR exp Acquired Immunodeficiency Syndrome/ or **acquired** deficiency virus.mp. OR acquired immune-deficiency.mp. or exp AIDS-Related Opportunistic Infections/ OR AIDS.mp. or exp Acquired Immunodeficiency Syndrome/ OR acquired	463999

		immun*.mp. OR deficiency syndrome.mp. OR sexually transmitted diseases, viral/ or exp hiv infections/	
	2. Candidiasis	candidiasis.mp. OR exp Candidiasis, Chronic Mucocutaneous/ or Candidiasis/ or exp Candidiasis, Oral/ OR thrush.mp. OR candidiosis.mp. OR candida infect*.mp. OR oral candidiasis.mp. OR candidi*.mp. OR Candida/ or candida.mp.	87375
	3. Oropharyngeal	oropharyngeal.mp. OR oral disease*.mp. OR orophyarynx.mp.	23688
	4. Randomized control trials	exp Randomized Controlled Trials as Topic/ OR Clinical Trials as Topic/ or Double-Blind Method/ or controlled clinical trial*.mp. or Randomized Controlled Trials as Topic/ OR clinical trial.mp. or Clinical Trial/ OR random allocation.mp. or *Random Allocation/ OR single blind method.mp. or Single-Blind Method/ OR research design.mp. OR comparative stud*.mp. OR *Prospective Studies/ or prospective stud*.mp. OR exp Evaluation Studies as Topic/ or evaluation stud*.mp.	4243433
	5.	# 2 AND #3	1398
	6.	#1 AND #5	608
	7.	#4 AND #6	251
**Embase on Ovid**	1. HIV	HIV.mp. or exp Human immunodeficiency virus/ OR HIV infections.mp. or Human immunodeficiency virus infection/ OR hiv-1.mp. or exp Human immunodeficiency virus 1/ OR hiv-2.mp. or exp Human immunodeficiency virus 2/ OR exp Human immunodeficiency virus/ or human immunodeficiency virus.mp. OR human immune deficient virus.mp. OR (human immun*.mp. AND deficiency virus.mp.) OR exp acquired immune deficiency syndrome/ or acquired immunodeficiency virus.mp. or exp Human immunodeficiency virus infection/ OR acquired immune deficiency.mp. OR AIDS.mp. or exp acquired immune deficiency syndrome/ OR (acquired immun*.mp. AND deficiency syndrome.mp.) OR sexually transmitted diseases,viral.mp. or sexually transmitted disease/	636468
	2. Candidiasis	candidiasis, oral.mp. or exp thrush/ OR candidiosis.mp. OR candida infect*.mp. OR candidi*.mp. OR exp Candida/ or candida.mp.	140492
	3. Oropharyngeal	oropharyngeal.mp. OR oral disease.mp. or exp mouth disease/ OR oropharynx.mp. or exp oropharynx/ or exp oropharynx candidiasis	650314
	4. Randomized control trial	Randomized Controlled Trial.mp. or exp randomized controlled trial/ OR clinical trial.mp. or exp clinical trial/ OR double blind procedure/ or double blind.mp. OR controlled clinical trial.mp. or exp controlled clinical trial/ OR single blind.mp. or exp single blind procedure/ OR random allocation.mp. or exp randomization/ OR exp placebo/ or placebo.mp. OR research design.mp. or exp methodology/ OR exp comparative study/ or comparative stud*.mp. OR exp prospective study/ or prospective stud*.mp. OR exp evaluation study/ or evaluation stud*.mp.	8225167
	5.	#2 AND #3	15753
	6.	#1 AND #5	3719
	7.	# 4 AND #6	1071
**Scopus**		(((TITLE-ABS-KEY ("HIV" OR "hiv-1" OR "hiv-2" OR "human immunodeficiency virus" OR "human immune deficiency virus" OR "human immune-deficiency virus" OR "HIV infections")) OR (TITLE-ABS-KEY ("acquired immune deficiency" OR "acquired immune deficiency syndrome" OR "acquired immunodeficiency syndrome" OR "acquired immunodeficiency" OR "AIDS" OR "AIDS related complex" OR "AIDS defining illnes*" OR "opportunistic infection*"))) AND (TITLE-ABS-KEY ("candidiasis" OR " candidiosis" OR "candida" OR "candidiasis infect*" OR "candidiosis infect*" OR "thrush" OR "oral candidiasis" OR	136

		"candidi*")) AND (TITLE-ABS-KEY ("oropharyngeal" OR "oropharynx" OR "oral lesion" OR "oral manifestation" OR "oral diseas*"))) AND (TITLE-ABS-KEY ("randomised control trial*" OR "randomized control trial*" OR "double blin*" OR "single blin*" OR "comparative" OR "placebo"))	
**CENTRAL**	1. HIV	Human immunodeficiency virus.mp. or exp HIV/ OR acquired immunodeficiency syndrome/ or aids-related complex/ or aids-related opportunistic infections/ OR AIDS.mp. OR HIV Infections/ or AIDS-Related Opportunistic Infections/ or Candidiasis, Oral/	26936
	2. Oropharyngeal	oral lesion*.mp. OR oropharynx.mp. or Oropharynx/ OR oropharyngeal.mp. OR oral manifestation*.mp.	4554
	3. Candidiasis	candidiasis.mp. or Candidiasis, Oral/ or Candidiasis OR Candida/ or candida infect*.mp.	2628
	4.	#1 AND #2 AND #3	103

- *** MESH terms # grouping**

Table S3. Studies excluded from quantitative analysis.

No	Author, Year	Title	Reason for Rejection
1	Goldman M et al, 2005 [27]	A randomized study of the use of fluconazole in continuous versus episodic therapy in patients with advanced HIV infection and a history of oropharyngeal candidiasis: AIDS Clinical Trials Group Study 323/Mycoses Study Group Study 40	Ineligible outcome (episodes)
2	MacPhail LA et al, 1996 [15]	Prophylaxis with nystatin pastilles for HIV-associated oral candidiasis.	Ineligible outcome (Outcome different
3	Nittayananta W et al, 2008 [28]	A randomized clinical trial of chlorhexidine in the maintenance of oral candidiasis-free period in HIV infection	Ineligible outcome (Outcome expressed as time to develop recurrent OPC)

Table S4. SUCRA rank of antifungal agents in the prevention of OPC among HIV-infected adults.

Intervention	All RCTs		
	**RR [95% CI]**	***p*-value**	**SUCRA Rank**
**Fluconazole**	0.45 [0.27, 0.77]	<0.05	1
**Placebo**	Reference		2
**Itraconazole**	1.11[0.34, 3.63]	>0.05	3
**Overall Inconsistency Chi-Square (*p* value)**	0.03(0.86)		
**Number of Studies**	**7**		

Table S5. Sensitivity analysis on primary efficacy outcome.

Intervention	Primary Analysis with All RCTS		RCTS with Low ROB	
	RR [95% CI]	SUCRA Rank	RR [95% CI]	SUCRA Rank
**Fluconazole**	0.45 [0.27, 0.77]	1	0.33 [0.14, 0.77]	1
**Placebo**	REFERENCE	2	REFERENCE	2
**Itraconazole**	1.11[0.34, 3.63]	3	1.11 [0.25, 4.79]	3
**Overall Inconsistency Chi-Square (*p* value)**	0.03(0.86)		0.02 (0.88)	
**Number of Studies**	7		5	

Table S6. General adverse effects reported (prevention of OPC).

Author (year)	Study Comparison	Percentage of Participants (%)
	Fever	
Stevens DA et al (1991)[31]	Fluconazole	8.3
	Placebo	7.7
	Night sweats	
Stevens DA et al (1991)[31]	Fluconazole	8.3
	Placebo	7.7
	Joint pain	
Stevens DA et al (1991)[31]	Fluconazole	0.0
	Placebo	7.7
	Neck swelling	
Stevens DA et al (1991)[31]	Fluconazole	8.3
	Placebo	0.0
	Adenopathy	
Stevens DA et al (1991)[31]	Fluconazole	8.3
	Placebo	0.0
	Headache	
Stevens DA et al (1991)[31]	Fluconazole	25.0
	Placebo	23.1

Table S7. Gastrointestinal adverse effects reported (prevention of OPC).

Author (year)	Study Comparison	Percentage of Participants (%)
Stevens DA et al (1991)[31]	Fluconazole	66.7
	Placebo	38.5
McKinsey DS et al (1999)[16]	Itraconazole	18.8
	Placebo	13.0

Table S8. Respiratory adverse effects reported (prevention of OPC).

Author (year)	Study Comparison	Percentage of Participants (%)
Stevens DA et al (1991)[31]	Fluconazole	33.3

	Placebo	30.8

Table S9. Dermatological adverse effects reported (prevention of OPC).

Author (Year)	Study Comparison	Percentage of Participants (%)
Just-Nubling G et al (1991)[30]	Fluconazole	2.3
	Untreated	0
Stevens DA et al (1991)[31]	Fluconazole	8.3
	Placebo	7.7
McKinsey DS et al (1999)[16]	Itraconazole	11.4
	Placebo	2.1

Table S10. Incidence of elevated liver enzymes reported (prevention of OPC).

Author (Year)	Study Comparison	Percentage of Participants (%)
Stevens DA et al (1991)[31]	Fluconazole	83.3
	Placebo	46.2
McKinsey DS et al (1999)[16]	Itraconazole	4.7
	Placebo	2.7

Table S11. SUCRA rank of the safety of antifungal agents used for the prevention of OPC among HIV-infected adults.

Intervention	All RCTs		
	RR [95% CI]	*p*-value	SUCRA rank
**Placebo**	Reference		1
**Fluconazole**	1.53 [1.02, 2.29]	<0.05	2
**Itraconazole**	1.96[1.16, 3.31]	<0.05	3
**Overall inconsistency Chi-square (*p* value)**	6.37 (0.062)		
**Number of studies**	7		

Table S12. Inconsistency in network meta-analysis.

Network Outcome	Chi-Square	*P* value for Test of Global Inconsistency
Prophylaxis: Incidence of OCP relapse	0.03	0.86
Prophylaxis: ADR	6.37	0.62

Table S13. Grade quality assessment.

Comparison	Direct Evidence		Indirect Evidence		Network Meta-Analysis	
	Risk Ratio (95% CI)	Quality of Evidence	Risk Ratio (95% CI)	Quality of Evidence	Risk Ratio (95% CI)	Quality of Evidence
**FLC vs PLC**	0.48(0.32,0.71)	Moderate *Ł	-	-	0.45 (0.27,0.77)	Moderate

**ITC vs PLC**	1.11(0.58,2.14)	Moderateƚ	-	-	1.11 (0.34,3.63)	Moderate
**FLC vs ITC**	-	-	-	Not estimatableŧ	0.41 (0.11,1.49)	Moderate

*Limitations (risk of bias). ȽLarge effect. ŧ No triangular or quadratic loops, not possible to generate indirect estimates using node splitting technique.
Abbreviations: A: Fluconazole; B: Itraconazole, C: Placebo

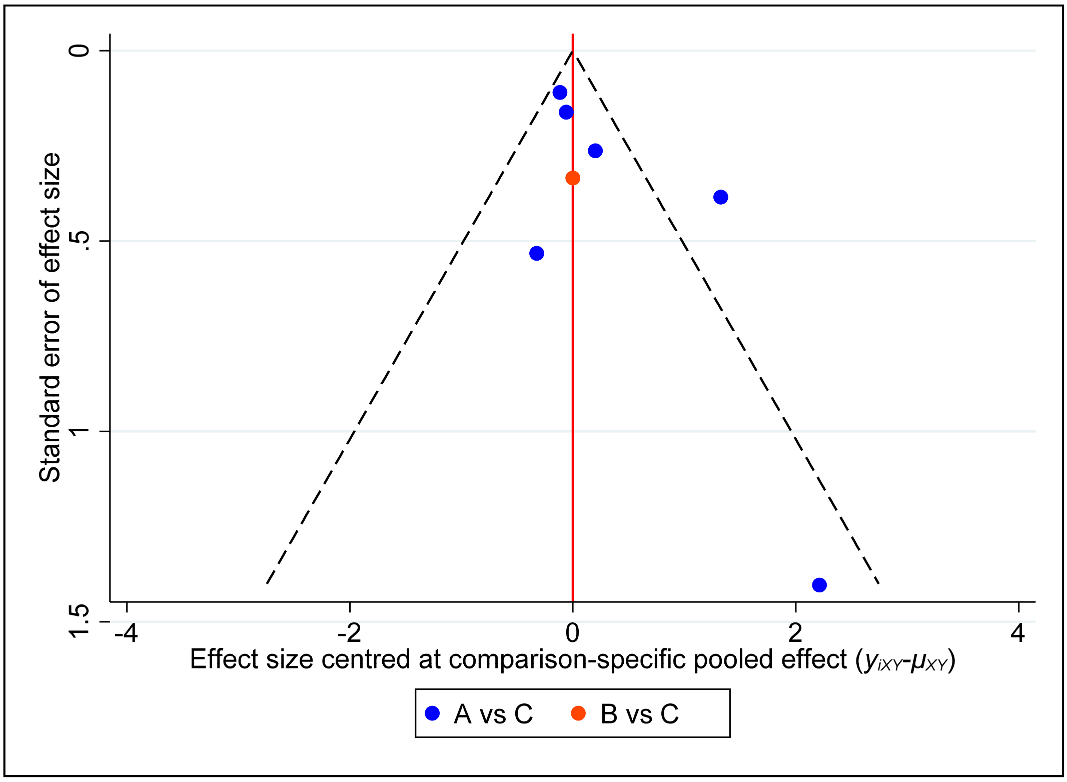

Abbreviations: A- Fluconazole, B- Itraconazole, C- Placebo.
Figure S1: Comparison-adjusted funnel plot of antifungal agents used for the prevention of OPC among HIV-infected adults (efficacy).

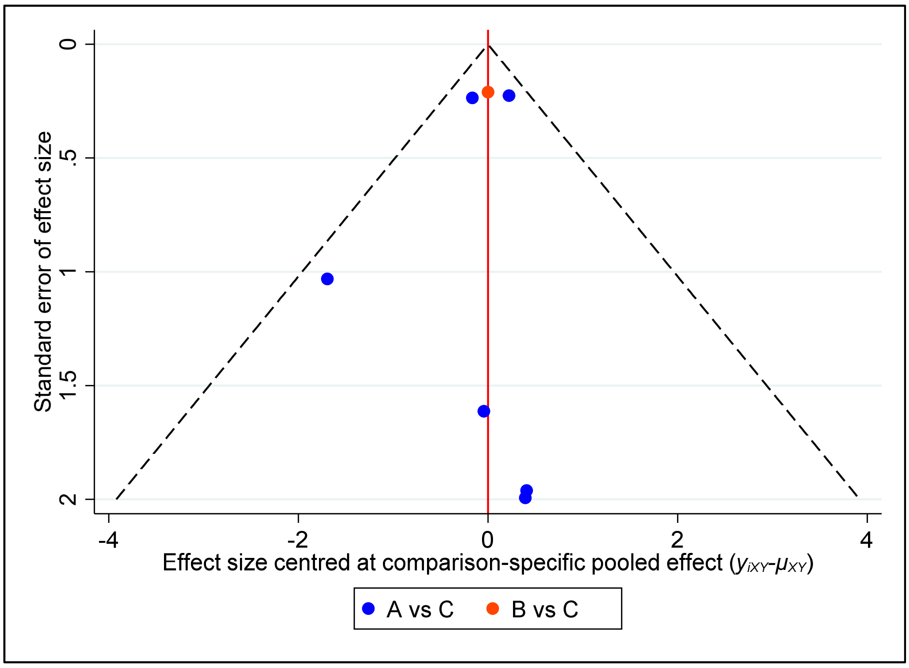

Abbreviations: A- Fluconazole, B- Itraconazole, C- Placebo.

Figure S2. Comparison-adjusted funnel plot of interventions used for the prevention of OPC among HIV-infected adults (safety profile).
